# Efficient Transdermal Delivery of Alendronate, a Nitrogen-Containing Bisphosphonate, Using Tip-Loaded Self-Dissolving Microneedle Arrays for the Treatment of Osteoporosis

**DOI:** 10.3390/pharmaceutics9030029

**Published:** 2017-08-17

**Authors:** Hidemasa Katsumi, Yutaro Tanaka, Kaori Hitomi, Shu Liu, Ying-shu Quan, Fumio Kamiyama, Toshiyasu Sakane, Akira Yamamoto

**Affiliations:** 1 Department of Biopharmaceutics, Kyoto Pharmaceutical University, Yamashina-ku, Kyoto 607-8414, Japan; tanaka.yutaro.27e@st.kyoto-u.ac.jp (Y.T.); lalala-strawbery-rip.pinky26@docomo.ne.jp (K.H.); liushu8852@gmail.com (S.L.); quan@cosmed-pharm.co.jp (Y.Q.); sakane@kobepharma-u.ac.jp (T.S.); yamamoto@mb.kyoto-phu.ac.jp (A.Y.); 2 CosMED Pharmaceutical Co., Ltd, Minami-ku, Kyoto 601-8014, Japan; kamiyama@cosmed-pharm.co.jp; 3 Department of Pharmaceutical Technology, Kobe Pharmaceutical University, Higashinada-ku, Kobe 658-8558, Japan

**Keywords:** self-dissolving microneedle arrays, dip-coating method, alendronate, transdermal absorption, skin irritation, osteoporosis

## Abstract

To improve the transdermal bioavailability and safety of alendronate (ALN), a nitrogen-containing bisphosphonate, we developed self-dissolving microneedle arrays (MNs), in which ALN is loaded only at the tip portion of micron-scale needles by a dip-coating method (ALN(TIP)–MN). We observed micron-scale pores in rat skin just after application of ALN(TIP)–MN, indicating that transdermal pathways for ALN were created by MN. ALN was rapidly released from the tip of MNs as observed in an in vitro release study. The tip portions of MNs completely dissolved in the rat skin within 5 min after application in vivo. After application of ALN(TIP)–MN in mice, the plasma concentration of ALN rapidly increased, and the bioavailability of ALN was approximately 96%. In addition, the decrease in growth plate was effectively suppressed by this efficient delivery of ALN in a rat model of osteoporosis. Furthermore, no skin irritation was observed after application of ALN(TIP)–MN and subcutaneous injection of ALN, while mild skin irritation was induced by whole-ALN-loaded MN (ALN–MN)—in which ALN is contained in the whole of the micron-scale needles fabricated from hyaluronic acid—and intradermal injection of ALN. These findings indicate that ALN(TIP)–MN is a promising transdermal formulation for the treatment of osteoporosis without skin irritation.

## 1. Introduction

Alendronate (ALN), a nitrogen-containing bisphosphonate, is effective in the treatment of bone diseases, including Paget’s disease, malignant hypercalcemia and osteoporosis [1]. The oral route has been the primary mode of ALN administration. Because ALN causes intestinal mucosal damage and its intestinal absorption is limited due to high polarity [2,3], an alternative delivery route for ALN is highly desirable, especially for bedridden elderly patients with bone disease.

Of the various strategies available, the transdermal route has received significant attention. The stratum corneum in the outermost layer of the skin restricts the skin penetration of hydrophilic and macromolecular drugs [4,5]. In order to solve this problem, a number of methods to improve transdermal drug transport have been evaluated, including chemical absorption enhancers [6], iontophoresis [7], sonophoresis [8], electroporation [9,10] and microneedle arrays (MN) [11]. Among these techniques, MN is an effective way to deliver macromolecular and hydrophilic drugs into systemic circulation from the skin.

Some of the candidate MN components include silicon, metals and polymers [12,13,14]. The transdermal absorption and bioavailability (BA) of various drugs were dramatically improved by MNs fabricated from these ingredients. Micron-scale needles of these MNs, however, are easy to break and may remain in the skin, leading to adverse effects. It is therefore desirable to fabricate MNs using biocompatible polymer-encapsulating agents for dissolution in the skin after application of the MN [15,16,17,18].

We have recently developed a self-dissolving MN, in which ALN is contained in the whole of the micron-scale needles fabricated from hyaluronic acid (ALN–MN) [19]. In this study, the BA of ALN after the application of ALN–MN was approximately 90% in rats. We also found, however, that ALN induced light erythema of the skin 4 days after removal of ALN–MN. This was probably because ALN loaded onto the base portion of the micron-scale needles remained in the skin after the application of ALN–MN. Therefore, we postulated that loading ALN only at the tip portion of micron-scale needles may cause drug release only at the dermis and rapid absorption from the skin, leading to the reduction or prevention of ALN-induced skin damage following application. Furthermore, the loading of ALN only on the needle tips allows for minimal drug loss. This delivery system may be a significant advance that enables efficient, safe and reliable transdermal administration in clinical use. Although tip-drug-loaded MNs have been reported previously [20,21,22,23], this method has not been applied for the transdermal delivery of bisphosphonates.

The aim of the present study was to develop a novel self-dissolving MN, in which ALN is loaded only at the tip portion of micron-scale needles, and to evaluate its transdermal absorption and safety after application in rodents. To this end, we designed a tip-ALN-loaded hyaluronic acid MN using a dip-coating method (ALN(TIP)–MN). The effect of ALN(TIP)–MN applied to the skin on skin damage was then evaluated in rats. We also investigated the absorption and therapeutic potential of ALN after transdermal application of ALN(TIP)–MN in rodents.

## 2. Materials and Methods

### 2.1. Materials

Hyaluronic acid was purchased from Kikkoman Biochemifa Company (Tokyo, Japan). ALN sodium trihydrate was obtained from Toronto Research Chemicals, Inc. (North York, ON, Canada). Paraformaldehyde, hematoxylin, eosin, and trypan blue were purchased from Wako Pure Chemical Industries, Ltd. (Osaka, Japan). Tissue-Tek^®^ O.C.T. compound was purchased from Sakura Finetek Japan Company, Ltd. (Tokyo, Japan). Pentobarbital sodium was purchased from Kyoritsu Seiyaku Co., Ltd. (Tokyo, Japan). Penicillin G and heparin sulfate were purchased from Nakalai Tesque Inc., (Kyoto, Japan). Absorbent gauze and adhesive plaster were purchased from Nichiban Company, Ltd. (Tokyo, Japan). All other chemicals were of the highest grade commercially available.

### 2.2. Animals

Male Wistar rats (8 weeks old, 220–250 g) and female Sprague–Dawley (S.D.) rats (7 weeks old, 200–220 g) were purchased from Japan SLC, Inc., (Shizuoka, Japan). Male HR-1 hairless mice (6 weeks old, 20–27 g) were purchased from Hoshino Laboratory Animals, Inc. (Ibaraki, Japan). These animals were maintained in conventional housing conditions with a daily 12 h light/dark cycle. Access to food and water was ad libitum. Animals were cared for in accordance with the National Institutes of Health Guidelines for the Care and Use of Laboratory Animals. The protocols for animal experiments were approved by the Animal Ethics Committee of the Kyoto Pharmaceutical University (Permit Number: 2012-077 and 2012-078).

### 2.3. Fabrication of ALN(TIP)-MN

The MNs were fabricated by micromolding technologies with hyaluronic acid as a base material, and were then loaded with 15 µg of ALN only at the tip portion of the micron-scale needles by a dip-coating method [20]. In brief, hyaluronic acid solution was put on micromolds of the MN at room temperature. After drying the micromold completely in a desiccator, the tip portion of the MN obtained by peeling the molds off the micromolds was dipped into 500 μL of 5% hyaluronic acid solution containing 15 μg ALN/MN within 2 s. After drying in a desiccator for 24 h at room temperature, ALN(TIP)–MN was obtained. Separately, ALN–MN, in which ALN is contained in the entire needle (800 μm in height) fabricated from hyaluronic acid, was also prepared as a control, as reported previously [19]. A digital microscope was used to inspect the completed arrays.

### 2.4. In Vitro Release of ALN from ALN(TIP)–MN

ALN(TIP)–MN was immersed in a glass vial containing 5 mL phosphate-buffered saline (PBS, pH 7.4), as reported previously [19]. In brief, the glass vial was maintained at 32 °C while stirred at 72 rpm. To determine the amount of ALN released from the ALN(TIP)–MN, the supernatant (0.3 mL) was drawn for analysis at predetermined times and was replaced with an equal volume of fresh PBS. All samples were stored at −20 °C until analysis, and the ALN concentration of each sample was determined by a reversed-phase high-performance liquid chromatography (RP–HPLC) method, as reported previously [24].

### 2.5. Dissolution of ALN(TIP)–MN after Application to the Rat Skin

Under pentobarbital anesthesia (50 mg/kg), ALN(TIP)–MN was applied to the dehaired abdomen of the rat. The MN was subsequently removed from the skin at 0, 5, 15, 30 and 60 min after the start of the experiment. A digital microscope was then used to visualize the dissolution of the ALN(TIP)–MN after the application to the abdominal skin of the rat [19].

### 2.6. Characterization of Rat Skin Pierced by ALN(TIP)–MN

The rat’s abdomen was shaved carefully using an animal clipper and shaver. Trypan blue solution (0.4%) was applied for 1 h to the skin surface of the MN insertion after a 5 min application of ALN(TIP)–MN to the abdominal skin of the rat. The stained skin was embedded in Tissue-Tek^®^ O.C.T. compound and subsequently fixed by plunge-freezing in liquid nitrogen. The frozen sample was stored at −80 °C and cryosectioned at −30 °C with a cryomicrotome (CM1100 R; LEICA Instruments, GmbH, Heidelberg, Germany) to produce skin sections of 10 µm thickness. The skin section was placed on a glass slide with a cover glass and examined using a microscope (BZ-8000; KEYENCE, Osaka, Japan) [19].

### 2.7. Transdermal Absorption Study

Under pentobarbital anesthesia (50 mg/kg), ALN(TIP)–MN was applied to the abdomen of HR-1 hairless mice at a dose of 1.5 mg ALN/kg (two pieces of MN containing 15 µg of ALN). Separately, ALN was orally administered to mice with a feeding needle at a dose of 50 mg/kg. ALN was also intravenously administered to mice via the tail vein at a dose of 1 mg/kg. At predetermined intervals, blood was collected from the caudal vena cava under isoflurane anesthesia. The plasma concentration of ALN was analyzed using an RP–HPLC method, as reported previously [24]. The peak ALN plasma concentration (*C*_max_) and time to reach *C*_max_ (*T*_max_) were determined directly from the plasma concentration–time profiles. The concentrations of ALN in plasma after intravenous injection were normalized with respect to μg/mL and analyzed using the nonlinear least-squares program MULTI [25]. The area under the concentration–time curve (AUC) after intravenous injection was calculated based on the two compartment model. The AUC after oral and transdermal administration was calculated by the trapezoidal formula and extrapolation to infinite time was based on a monoexponential equation [26].

### 2.8. Osteoporosis Experiment

To evaluate the preventive effect of ALN from ALN(TIP)–MN against osteoporosis, 12 female S.D. rats were randomized into 4 groups of 3 rats each and either sham-operated (sham, 1 group) or ovariectomized (OVX, 3 groups). OVX was performed under pentobarbital anesthesia (64.8 mg/kg) to establish a postmenopausal osteoporosis model [19,27]. Penicillin G (8 mg/mL) was then administered subcutaneously just after the operation. In the study on preventive effect, on the day of the operation, ALN(TIP)–MN (75 µg ALN/kg; 1 piece of MN containing 15 µg of ALN) was applied to the dehaired abdomen of the first group of OVX rats weekly for 24 h over 4 weeks. Separately, ALN was orally administered to the second group of OVX rats at a dose of 75 µg ALN/kg. The third group of OVX rats was maintained without administration of ALN. The right tibia was excised 4 weeks after OVX, fixed in 4% buffered paraformaldehyde and embedded in paraffin blocks; 5 µm-thick sections were cut from these paraffin blocks. The sections from the right tibia were stained with hematoxylin and eosin, and the bone morphology of the right tibia was characterized using a BIO-ZERO microscope (KEYENCE, Osaka, Japan).

Separately, the study on therapeutic effect was also examined. Four weeks after OVX, ALN was administered over 4 weeks, and subsequently the bone morphology of the right tibia was characterized 8 weeks after OVX, as described above.

### 2.9. Skin Irritation in Response to ALN(TIP)–MN

The male Wistar rat’s abdomen or back was shaved carefully using an animal clipper and shaver under pentobarbital anesthesia (50 mg/kg). ALN(TIP)–MN or ALN–MN, in which ALN is contained in the whole needles fabricated from hyaluronic acid (60 µg ALN/kg; 1 piece of MN containing 15 µg of ALN), was applied to the abdomen of the rat over a period of 24 h. Separately, 100 µL of ALN solution (60 µg ALN/kg) was administrated subcutaneously or intradermally to the back of the rat. The application sites of ALN were marked using a felt-tip pen, covered with absorbent gauze and fixed using adhesive bandages. The macroscopic appearance of the skin was recorded daily, before application of ALN(TIP)–MN and after its removal, over 7 days [19].

## 3. Results

### 3.1. Characteristics of ALN(TIP)–MN

**Figure 1:**
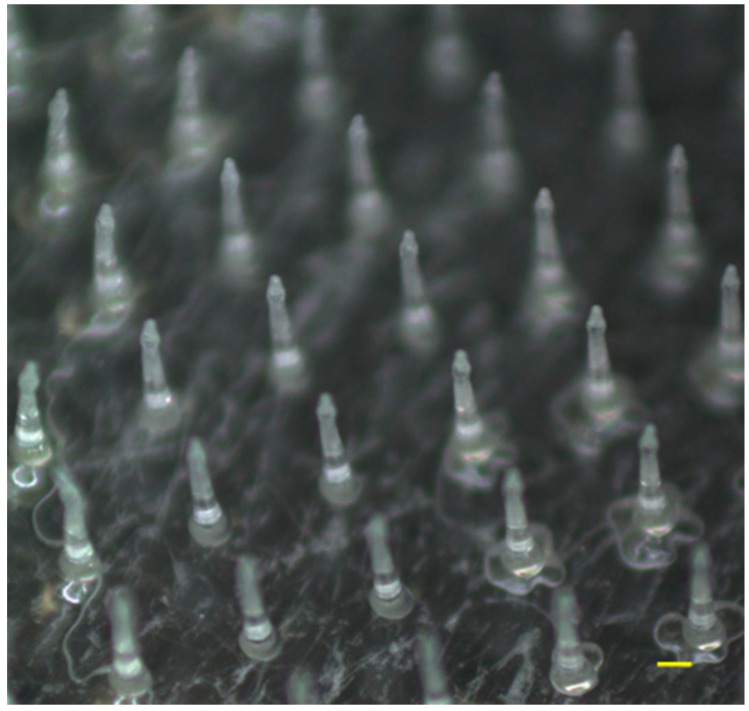
Micrograph of ALN(TIP)–MN (tip-ALN-loaded hyaluronic acid MN). Scale bar: 400 μm.

Figure 1 shows a micrograph of ALN(TIP)–MN using a digital microscope. The resulting tapered-cone MNs were uniform in size with sharp tips; each MN was approximately 800 µm in height, with a diameter of 160 µm at the base, 40 µm at the tip, and an interspacing of 600 µm between rows of the microneedle arrays. ALN was loaded onto the tips of the MN to a depth of about 200 μm. Each MN had approximately 190 needles within a portion that was 10 mm in diameter.

### 3.2. In Vitro Release Profile of ALN from ALN(TIP)–MN

**Figure 2:**
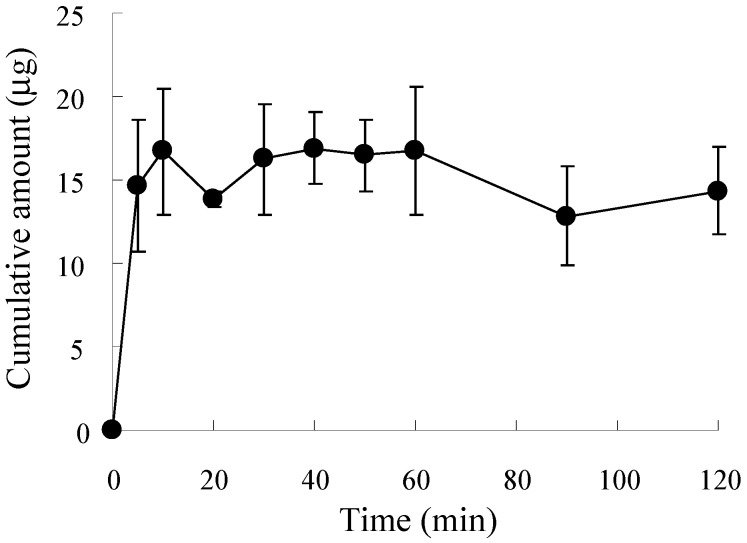
The release profile of ALN from ALN(TIP)–MN in phosphate buffered saline (PBS). Results are expressed as the mean ± S.E. of three experiments.

Figure 2 shows the release profile of ALN from ALN(TIP)–MN in PBS. The ALN (15 µg) loaded onto the needles was completely released from ALN(TIP)–MN by 5 min after addition to PBS.

### 3.3. Dissolution Process of ALN(TIP)–MN after Application to Rat Skin

**Figure 3:**
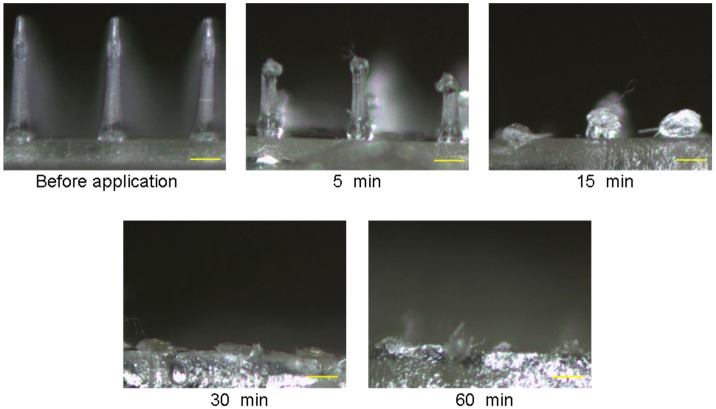
The dissolution process of ALN(TIP)–MN after the application to rat skin. Scale bar: 200 μm.

The dissolution profile of ALN(TIP)–MN in rat skin in vivo is shown in Figure 3. The tips of the needles completely dissolved within 5 min after application of ALN(TIP)–MN, and all of the needles completely dissolved within 30 min.

### 3.4. Piercing Ability of ALN(TIP)–MN across Rat Skin

**Figure 4:**
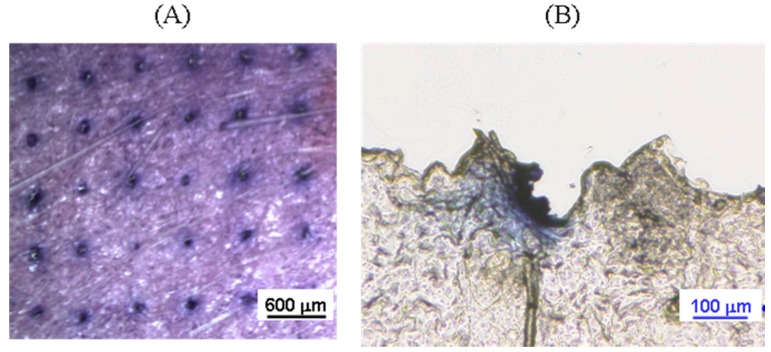
Photograph (**A**) and micrograph (**B**) of rat skin after application of ALN(TIP)–MN. Scale bar: (**A**) 600 μm, (**B**) 100 μm.

To study the piercing ability of ALN(TIP)–MN, the formation of the micron-sized pores after application of ALN(TIP)–MN was visualized based on the appearance of blue dots in the skin. Figure 4 shows skin surface and skin section images after application of ALN(TIP)–MN to rats. The micron-scale pores were created in the skin after a five-minute application of ALN(TIP)–MN, and the needles pierced the stratum corneum.

### 3.5. Pharmacokinetics of ALN after Various Administration Methods

**Figure 5:**
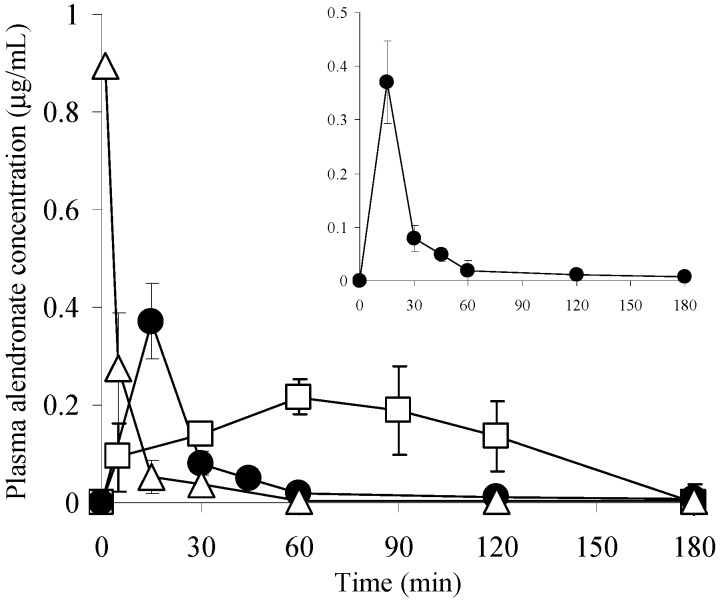
Plasma concentrations of ALN after various administration methods. Results are expressed as the mean ± S.E. of three mice. ●, ALN(TIP)–MN (1.5 mg ALN/kg); △, i.v. (1 mg ALN/kg); □, p.o. (50 mg ALN/kg). i.v.; intravenous injection, p.o.; oral administration.

Figure 5 shows the pharmacokinetics of ALN after application of ALN(TIP)–MN. The plasma ALN concentration rapidly decreased after intravenous injection, and the plasma ALN concentration gradually increased and reached 0.22 ± 0.04 μg/mL 60 min after oral administration of 50 mg/kg ALN. In contrast, the plasma ALN concentrations increased rapidly and reached the maximum concentration within 15 min after application of the ALN(TIP)–MN at a dose of 1.5 mg ALN/kg. Table 1 shows the pharmacokinetic parameters of ALN after various administration methods. The BAs of ALN were approximately 8.3% and 96%, after oral administration and ALN(TIP)–MN application, respectively.

### 3.6. Preventive and Therapeutic Effect of ALN(TIP)–MN on Osteoporosis

**Figure 6:**
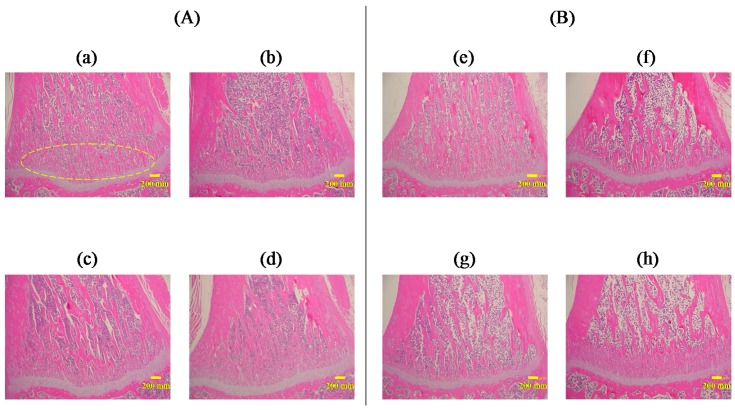
Histological micrographs of bone tissue from the right tibia of rats. (**A**) Preventive effect of ALN on the decrease in the width of the growth plate (circled area) and density of bone structure after administration. (a) Sham (sham-operated), (b) OVX (ovariectomized), (c) weekly oral administration of ALN just after OVX, (d) weekly application of ALN(TIP)–MN just after OVX. All groups were evaluated 4 weeks after sham or OVX operation. (**B**) Therapeutic effect of ALN on the decrease in the width of the growth plate and density of bone structure after administration. (e) Sham, (f) OVX, (g) weekly oral administration of ALN 4 weeks after OVX, (h) weekly application of ALN(TIP)–MN 4 weeks after OVX. All groups were evaluated 8 weeks after sham or OVX operation. Original magnification: 40×. Scale bar: 200 μm. Representative data are shown from one of three experiments.

Figure 6 shows the effect of ALN(TIP)–MN on the density and structure of bone in the osteoporosis model rats. In the study on preventive effects (Figure 6A), bone density decreased 4 weeks after OVX operation, indicating postmenopausal osteoporosis induction (Figure 6(Ab)). Oral administration of ALN hardly affected the decrease in the width of the growth plate and bone density induced by OVX (Figure 6(Ac)). In contrast, the decrease in the width of the growth plate was effectively prevented by application of ALN(TIP)–MN in OVX rats (Figure 6(Ad)). Similarly, in the study on therapeutic effects (Figure 6B), we found that ALN(TIP)–MN was more effective in the treatment of postmenopausal osteoporosis than oral administration of ALN.

### 3.7. Skin Irritation Caused by ALN after Various Administration Methods

**Figure 7:**
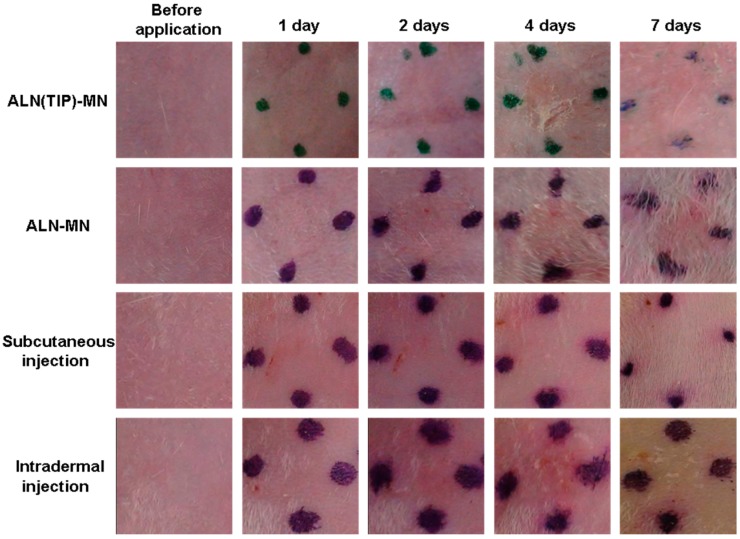
Skin irritation after administration of ALN with various methods. The macroscopic appearance of the skin was recorded daily before application of the ALN(TIP)–MN and after its removal over 7 days. The area surrounded with points shows the site where ALN was administered. Representative data are shown from one of three experiments.

Figure 7 shows our observations of the rat skin surface after administration of ALN using various methods. Light erythema was observed 4 days after application of ALN–MN, in which ALN is contained in the whole needles fabricated from hyaluronic acid, and intradermally injected. In contrast, no skin irritation was observed for 7 days after administration of ALN(TIP)–MN and subcutaneous injection of ALN.

## 4. Discussion

In the present study, the transdermal absorption of ALN occurred without skin irritation by loading ALN onto the microneedle tips fabricated from hyaluronic acid. Hyaluronic acid is biocompatible, highly water soluble and has been widely used in cosmetics [28]. MNs, fabricated from hyaluronic acid, were therefore easily dissolved in the skin and can be applied without skin irritation [20,29,30]. In the present study, the rapid dissolution of the tip of the MN in the skin indicated that the loaded ALN can be rapidly released in the skin after application. The creation of micron-scale pores in the skin after application of ALN(TIP)–MN showed that the MNs fabricated from hyaluronic acid have sufficient mechanical strength to penetrate the skin, even though a high concentration of ALN was loaded at the tip portion of the micron-scale needles. Although the depth of the created pores on the skin was shorter than the needle length, these findings were in agreement with previous results, which indicated that the created pores quickly recovered after application of normal MNs, in which drugs were loaded onto whole needles fabricated from hyaluronic acid [29,30]. In general, the mean thickness of the stratum corneum and epidermis of the skin is approximately 10–20 and 70–100 µm, respectively [31]. Therefore, ALN(TIP)–MN (800 µm length) has a suitable length of needles required to reach the upper dermis under the stratum corneum and the epidermis. Because ALN was loaded on the first 200 µm at the top of the MN, ALN (TIP)–MN was able to deliver ALN directly to the dermis layer, where capillaries are located. In fact, the plasma ALN concentration increased promptly just after application of ALN(TIP)–MN in the present study. In our previous study, dissolving MNs, in which drugs were contained in the whole needles, showed high BA of ALN (over 90%), but the increase in plasma concentration tended to be delayed compared to that observed with subcutaneous injection [19,29]. These results indicated that rapid absorption can be obtained by loading ALN only on the needle tips.

The preventive and therapeutic effects of ALN(TIP)–MN on osteoporosis were proportional to the pharmacokinetics of ALN after the application of ALN(TIP)–MN. ALN inhibits bone resorption by suppressing osteoclast function after being distributed in bone [32]. These results suggest that ALN was released and absorbed promptly from ALN(TIP)–MN after application, and that ALN(TIP) –MN had efficient preventive and therapeutic effects on osteoporosis by suppressing osteoclast function.

To establish a pragmatic transdermal drug delivery system, minimal skin irritation on the site of the application is highly preferred. Bisphosphonates, including ALN, easily induce mucosal damages, including gastritis, gastric ulcers and erosive esophagitis after oral administration [33,34,35]. Some researchers have reported the mechanism of mucosal damage induced by nitrogen-containing bisphosphonates. Lichtenberger et al. reported that the displacement of bisphosphonates and zwitterionic phospholipids, such as phosphatidylcholine, within adherent mucus gel, increased the gastric mucosal susceptibility to acid attack [34]. Moreover, Reszka et al. reported that bisphosphonates inhibited the keratinocyte cell cycle in the esophagus [35]. Because epithelial and dermal cells in the skin have similar structures and functions as those in the upper gastrointestinal tract, ALN is thought to induce skin irritation after transdermal administration. We previously observed mild and protracted skin irritation using an ALN patch prepared with hydrophilic adhesive and ALN–MN, in which ALN was contained in the whole needles fabricated from hyaluronic acid [19]. This is probably because ALN remained in the epidermis for a long period of time after administration of ALN to the skin. The present study, on skin irritation after the application of two types of MNs and various injection methods, indicated that loading ALN only on the microneedle tips could decrease the ALN distribution in the epidermis and increase the elimination of ALN from the dermis to the blood stream, leading to the avoidance of ALN-induced skin irritation. These results indicate that the direct delivery of ALN to the dermis is a promising approach for the avoidance of ALN-induced skin irritation.

## 5. Conclusions

In conclusion, we successfully developed ALN(TIP)–MN, which has ALN only at the tip portion of micron-scale needles. ALN(TIP)–MN provided rapid absorption of ALN and an efficient preventive and therapeutic effect on osteoporosis. Furthermore, ALN-induced skin irritation can be avoided by direct delivery of ALN to the dermis using tip-drug-loaded MNs. These findings indicate that tip-drug-loaded MNs are a promising formulation for the efficient and safe transdermal delivery of ALN in patients undergoing treatment for osteoporosis.

## Figures and Tables

**Table 1:** Pharmacokinetic parameters of ALN (alendronate) after various administration methods.

	Dose (mg/kg)	*C*_max_ (μg/mL)	*T*_max_ (min)	AUC (μg·min/mL)	BA (%)
i.v.	1	-	-	5.64	-
p.o.	50	0.22 ± 0.04	60	23.5	8.3
ALN(TIP)–MN	1.5	0.37 ± 0.08	15	8.16	96

*C*_max:_ maximum plasma concentration, *T*_max_: time to maximum plasma concentration, AUC: area under the plasma concentration-time curve, BA: bioavailability, i.v.: intravenous injection, p.o.: oral administration.
